# Author reply in response to a letter on “High-flow nasal oxygen cannula vs. noninvasive mechanical ventilation to prevent reintubation in sepsis: a randomized controlled trial”

**DOI:** 10.1186/s13613-021-00964-9

**Published:** 2021-12-13

**Authors:** Surat Tongyoo, Porntipa Tantibundit, Kiattichai Daorattanachai, Tanuwong Viarasilpa, Chairat Permpikul, Suthipol Udompanturak

**Affiliations:** 1 Division of Critical Care Medicine, Department of Medicine, Faculty of Medicine, Siriraj Hospital, Mahidol University, No. 2, Prannok Road, Bangkoknoi, Bangkok, 10700 Thailand; 2 Department of Emergency Medicine, Khon Kaen Hospital, Khon Kaen, Thailand; 3 Department of Emergency Medicine, Faculty of Medicine, Thammasat University, Pathum Thani, Thailand; 4 Office of Research and Development, Faculty of Medicine, Siriraj Hospital, Mahidol University, Bangkok, Thailand

Dear Editor,

We appreciate the valuable comments from Toumi et al. regarding our article “High-flow nasal oxygen cannula vs. noninvasive mechanical ventilation to prevent reintubation in sepsis: a randomized controlled trial” [1] and thank you for the opportunity to respond to their concerns. The following is our author response.

We consider the concern of the absence of pulmonary mechanics information in our reported study, which may cause the reader to be unable to identify the type of patients who might benefit from noninvasive mechanical ventilation (NIV) over high-flow nasal oxygen cannula (HFNC) and vice versa. In this response letter, we provide the patients’ airway pressures, tidal volume, respiratory system compliance and airway resistance, which were measured before extubation, in Table 1. For respiratory system compliance and airway resistance measurement, we switched mechanical ventilator mode from pressure support ventilation (PSV) to volume-controlled (VC) mode for a short period. During VC mode, we set tidal volume equal to the average tidal volume of the patient during ventilated under PSV mode. The inspiratory flow rate was set at 60 L per minute with the square wave pattern. The respiratory rate was set about 70% of the patient’s respiratory rate during PSV. Then we set an inspiratory hold for 0.5 s. The plateau pressure was measured at the end of inspiratory hold. The peak airway pressure was measured at the highest inspiratory pressure. We did not change the positive end expiratory pressure (PEEP) and FiO_2_ during plateau pressure measurement. There was no significant difference among all pulmonary mechanic parameters between NIV and HFNC. This could be the result of the blind-randomization process, wherein we keep the device assignment in a closed envelope until the patient was enrolled with a signed informed consent and ready to extubate. Then the sealed envelope was allowed to be opened. This process limited the device selection bias and allows us to enrol well-matched patients in the NIV and HFNC groups. Furthermore, our study assessed the readiness for weaning and extubation according to the standard guideline, by using the clinical assessment and several objective parameters. Therefore, the pulmonary mechanics before enrolment and extubation showed that at least 75% of patients in both groups had respiratory system static compliance and airway resistance within normal range [2] (Table 1). The observation of high respiratory system compliance among our patients could be explained by the resolution of lung pathology after treatment. Another possibility could be that during weaning, our patients were free from the paralytic agent; hence, the patient’s inspiratory effort could interfere with the plateau pressure measurement and resulting in an overestimation of respiratory system compliance. With this additional pulmonary mechanic data and information from the previously presented subgroup analysis, we did not identify any specific condition that prefers NIV over HFNC nor vice versa, in the prevention of reintubation among sepsis patients.

**Table 1:** Pulmonary mechanic parameters of the patients

Pulmonary mechanic parameters, median (IQR)	NIV (*N* = 110)	HFNC (*N* = 112)	*P*
Peak inspiratory pressure (cmH_2_O)	16 (14–18)	16 (14–20)	0.38
Plateau pressure (cmH_2_O)	12 (10–14)	12 (10–15)	0.59
Positive end expiratory pressure (PEEP, cmH_2_O)	5 (5–6)	5 (5–5)	0.45
Tidal volume (mL/kg)	7.1 (5.7–8.6)	7.1 (6.0–8.8)	0.99
Fraction of inspired oxygen (FiO_2_, %)	40 (40–40)	40 (40–50)	0.45
Respiratory system static compliance^a^ (mL/cmH_2_O)	80 (50–130)	70 (50–100)	0.14
Airway resistance^b^ (cmH_2_O/L/s)	4 (4–5)	4 (4–5)	0.48

*NIV* noninvasive ventilation, *HFNC* high-flow nasal oxygen cannular, *SD* standard deviation, *IQR* interquartile range: *cmH*_*2*_*O* centimetre of water, *mL/kg* millilitre per kilogramme, *mL/cmH*_*2*_*O* millilitre per centimetre of water, *cmH*_*2*_*O/L/sec* centimetre of water per litre per second, *ABG* arterial blood gas analysis, *PaO*_*2*_ partial pressure of oxygen in arterial blood, *PaCO*_*2*_ partial pressure of carbon dioxide in arterial blood, *FiO*_*2*_ fraction of inspired oxygen, *mmHg* millimetre of mercury

^a^Normal respiratory system static compliance is 60–100 mL/cmH_2_O

^b^Normal airway resistance is less than 5 cmH_2_O/L/s

Psychological dysfunction and neuromuscular incompetence were documented as one of the pathophysiological factors of weaning failure [2–4]. In this study, we enrolled the patients who pass the daily assessment of weaning readiness. Richmond Agitation–Sedation Scale (RASS) score was used as a part of weaning readiness assessment [4]. The patient, who had RASS ≥ − 1 without neuromuscular blocking agent use in the last 12 h, was considered as weaning candidate, while those with RASS < − 1 were not candidates for weaning on that day. We also excluded the patients who developed agitation or altered mental status during spontaneous breathing trial. This meant that, our study participants were fully awake, with no agitation and had adequate respiratory muscle strength, before extubation. After extubation and applying the study devices, one patient in the NIV group and four patients in the HFNC group had newly developed altered mental status and required reintubation. This observation did not show any statistical difference between groups.

Finally, we totally agree with Toumi et al. that different means of ventilatory support should be adapted in accordance with the mechanism and severity of the post-extubation respiratory failure. This could be the standard treatment in daily clinical practice for the treatment of acute respiratory failure developed after extubation, especially when the direct clinical cause of the respiratory failure; for example, left heart failure, neuromuscular disorders, or delirium, was identified. However, it differs from the objective of our research, which aims to compare the efficacy of NIV versus HFNC in the prevention of post-extubation respiratory failure, before it occurs [1]. Further study, with different decide, would be needed to identify a subgroup of patients with the same mechanism of post-extubation respiratory failure that would benefit from one technique over the other.

## Data Availability

None.

## Abbreviations

HFNC: High-flow nasal cannula
NIV: Noninvasive ventilation
P-SILI: Patient Self-Inflicted Lung Injury
